# An essential role of SVZ progenitors in cortical folding in gyrencephalic mammals

**DOI:** 10.1038/srep29578

**Published:** 2016-07-12

**Authors:** Tomohisa Toda, Yohei Shinmyo, Tung Anh Dinh Duong, Kosuke Masuda, Hiroshi Kawasaki

**Affiliations:** 1 Department of Medical Neuroscience, Graduate School of Medical Sciences, Kanazawa University, Ishikawa 920-8640, Japan; 2 Brain/Liver Interface Medicine Research Center, Kanazawa University, Ishikawa 920-8640, Japan; 3 Department of Neurology, Graduate School of Medicine, The University of Tokyo, Tokyo 113-0033, Japan

## Abstract

Because folding of the cerebral cortex in the mammalian brain is believed to be crucial for higher brain functions, the mechanisms underlying its formation during development and evolution are of great interest. Although it has been proposed that increased neural progenitors in the subventricular zone (SVZ) are responsible for making cortical folds, their roles in cortical folding are still largely unclear, mainly because genetic methods for gyrencephalic mammals had been poorly available. Here, by taking an advantage of our newly developed *in utero* electroporation technique for the gyrencephalic brain of ferrets, we investigated the role of SVZ progenitors in cortical folding. We found regional differences in the abundance of SVZ progenitors in the developing ferret brain even before cortical folds began to be formed. When Tbr2 transcription factor was inhibited, intermediate progenitor cells were markedly reduced in the ferret cerebral cortex. Interestingly, outer radial glial cells were also reduced by inhibiting Tbr2. We uncovered that reduced numbers of SVZ progenitors resulted in impaired cortical folding. When Tbr2 was inhibited, upper cortical layers were preferentially reduced in gyri compared to those in sulci. Our findings indicate the biological importance of SVZ progenitors in cortical folding in the gyrencephalic brain.

Folding of the cerebral cortex is a distinctive feature of the mammalian brain. It has been believed that cortical folding underlies the acquisition of higher brain functions during development and evolution. In fact, loss of cortical folding (lissencephaly) severely affects intellectual abilities in humans^1^,^2^,^3^. It has been proposed that the acquisition of cortical folding during evolution resulted from increased neural progenitors because increased cell proliferation was found in the cerebral cortex of gyrencephalic mammals early in development^4^,^5^,^6^,^7^,^8^,^9^,^10^,^11^,^12^.

Neural progenitors in the cerebral cortex are organized in two germinal layers: the ventricular zone (VZ) and the subventricular zone (SVZ). The VZ consists of radial glial cells (RGs, also known as apical progenitors/ventricular RGs/apical RGs), the epithelial stem cells that line the cerebral ventricles and extend apical fibers and basal fibers. RGs in the VZ undergo multiple rounds of asymmetric cell divisions and generate SVZ progenitors. The SVZ is further subdivided into the outer SVZ (OSVZ) and the inner SVZ (ISVZ) and contains two types of SVZ progenitors: intermediate progenitor cells (IPCs, also known as basal progenitors), and the other is recently identified outer radial glial cells (oRGs, also known as OSVZ RGs/basal RGs/intermediate RGs/translocating RGs)^13^,^14^,^15^,^16^. IPCs delaminate from the VZ to form the SVZ, lose their apico-basal polarity, and generate daughter neurons^17^,^18^. oRGs also delaminate from the VZ, but they retain characteristics of RGs such as apico-basal polarity^13^,^14^,^15^.

Several studies demonstrated the existence of a prominent thick SVZ in a variety of gyrencephalic species including ferrets, cats, monkeys and humans. Since IPCs and oRGs are abundant in the SVZ of gyrencephalic mammals compared with that of lissencephalic rodents, it has been hypothesized that the increased IPCs and/or oRGs lead to cortical folding^13^,^14^,^15^,^19^. In contrast, recent reports also showed that despite lissencephalic cortical morphology, SVZ progenitors were observed with similar abundance in developing marmosets to those in the developing humans and ferrets^20^,^21^, raising another hypothesis that the increase of SVZ progenitors is dispensable for cortical folding.

These two hypotheses have not been addressed, mainly because *in vivo* genetic manipulations that can be applied to the cerebral cortex of gyrencephalic mammals had not been established. We therefore recently established a rapid and efficient genetic manipulation method for gyrencephalic carnivore ferrets using *in utero* electroporation (IUE)^22^,^23^. We demonstrated that neural progenitors in the cerebral cortex of developing ferrets could be efficiently transfected using our IUE technique^22^,^23^. Using our IUE technique, we recently demonstrated that ectopic expression of FGF8 in the ferret cerebral cortex led to an increase in SVZ progenitors and polymicrogyria, suggesting that increased SVZ progenitors may underlie the formation of additional gyri^24^. By taking an advantage of IUE, here we investigate the roles of SVZ progenitors in cortical folding in the ferret brain. We show that, when the T-domain transcription factor Tbr2 (also known as Eomes) is inhibited, not only IPCs but also oRGs are markedly decreased, and that the reduced numbers of SVZ progenitors result in impaired cortical folding. We further show that, when Tbr2 was inhibited, the thicknesses of upper cortical layers are preferentially reduced in gyri compared to those in sulci. Our results indicate the biological importance of SVZ progenitors in cortical folding.

## Results

### Regional difference of the abundance of SVZ progenitors in the ferret cerebral cortex during development

**Figure 1:**
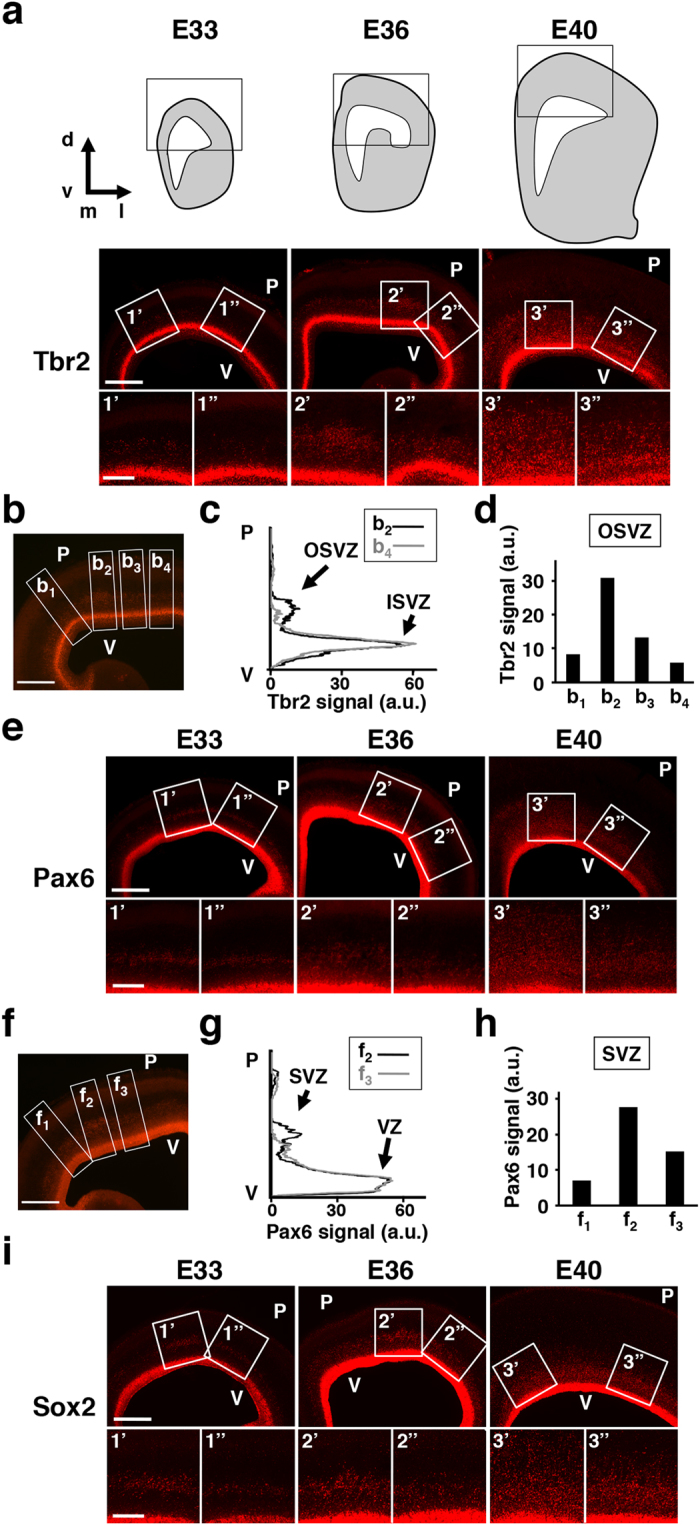
Regional difference of the abundance of SVZ progenitors in the ferret cerebral cortex during development. Immunostaining using the cerebral cortex of ferret embryos. The cerebral cortex was prepared at the indicated time points, and coronal sections were subjected to immunostaining for Tbr2 (**a**–**d**), Pax6 (**e**–**h**) and Sox2 (**i**). The areas within the boxes in (**a**,**e**,**i**) were magnified and are shown in the lower panels (1′, 1″, 2′, 2″, 3′ and 3″). ((**a**), top) The schematic drawings representing coronal sections we analyzed. Immunohistochemical images within the boxes are shown below. d, dorsal; v, ventral; m, medial; l, lateral. ((**a**), bottom) Tbr2-positive IPCs were more abundant in certain cortical areas (2′, 3′) than others (2″, 3″). (**b**) A representative image at E36 containing four ROIs (b_1_–b_4_) used for quantification of Tbr2 signal intensities. (**c**) Tbr2 signal intensities within the ROI b_2_ and b_4_ were measured along the radial axis and plotted against the distance from the ventricular surface. Note that Tbr2 signal intensities in the OSVZ were different between ROI b_2_ and b_4_, while those in the ISVZ were almost the same. (**d**) Tbr2 signal intensities in the OSVZ of four ROIs (b_1_–b_4_). (**e**) Pax6-positive oRGs were more abundant in certain cortical areas (2′, 3′) than others (2″, 3″). (**f**) A representative image at E36 containing three ROIs (f_1_–f_3_) used for quantification of Pax6 signal intensities. (**g**) Pax6 signal intensities within the ROI f_2_ and f_3_ were measured along the radial axis and plotted against the distance from the ventricular surface. Note that Pax6 signal intensities in the SVZ were different between ROI f_2_ and f_3_, while those in the VZ were almost the same. (**h**) Pax6 signal intensities in the SVZ of three ROIs (f_1_–f_3_). (**i**) Sox2-positive oRGs were more abundant in certain cortical areas (2′, 3′) than others (2″, 3″). V, ventricular surface; P, pial surface. Scale bars = 500 μm ((**a**,**e**,**i**,) upper; (**b**,**f**)) and 100 μm ((**a**,**e**,**i**,) lower).

We first examined the spatial distribution patterns of SVZ progenitors during development in the ferret cerebral cortex. We performed immunostaining for the IPC marker Tbr2 and the oRG markers Pax6 and Sox2^14^,^15^. As reported previously^14^,^15^,^25^, Tbr2-positive IPCs, Pax6- and Sox2-positive oRGs increased gradually from E33 to E40 (Fig. 1a,e,i). Interestingly, the distribution patterns of SVZ progenitors were not uniform throughout the cerebral cortex: Tbr2-positive IPCs and Pax6- and Sox2-positive oRGs in the OSVZ were more abundant in certain cortical areas than in other cortical areas (Fig. 1a,e,i; compare 2′ vs. 2″ at E36, and 3′ vs. 3″ at E40). We then quantified Tbr2 signal intensities along four different radial axes in the cerebral cortex at E36 (Fig. 1b,b1–b4). Tbr2 signals indeed showed regional differences in the OSVZ (Fig. 1c,d), where as those in the ISVZ were almost comparable between regions (Fig. 1c). Pax6 signal intensities also showed regional differences in the SVZ (Fig. 1f–h), where as those in the VZ were almost the same between regions (Fig. 1g).

These regional differences in the abundance of IPCs and oRGs in the SVZ were not obvious at E33, became detectable at E36 and were more prominent at E40 (Fig. 1a,e,i). Because the distribution of SVZ progenitors in the lissencephalic rodent cortex is almost uniform during cortical development^26^,^27^, these findings suggest that not only the increased numbers of SVZ progenitors, but also the regional differences in the abundance of SVZ progenitors are important features of the gyrencephalic mammalian cortex. It is worth noting that this regional difference in the abundance of SVZ progenitors appears before cortical folds are formed during development. Cortical folds are invisible at the birth of pups (i.e. E42) and are formed in the first postnatal month in ferrets. These observations support the hypothesis that SVZ progenitors are responsible for cortical folding^4^,^9^,^11^,^25^,^28^.

It would be intriguing to examine whether areas containing abundant SVZ progenitors corresponds to presumptive areas of gyrification. However, cortical folds appear postnatally (i.e. the 2nd week after birth) in ferrets, and it was difficult to test this point using the embryonic ferret brain. We therefore examined the expression patterns of Pax6 and Tbr2 at P6, when cortical folds are about to be formed. Our immunohistochemical analyses showed that the areas containing abundant SVZ progenitors at P6 correspond to the areas that would develop gyri (Supplementary Fig. 1). These results were consistent with a previous work showing the distribution patterns of Tbr2 in ferrets^25^.

### Essential roles of Tbr2 in the production of both IPCs and oRGs in the developing ferret cortex

**Figure 2:**
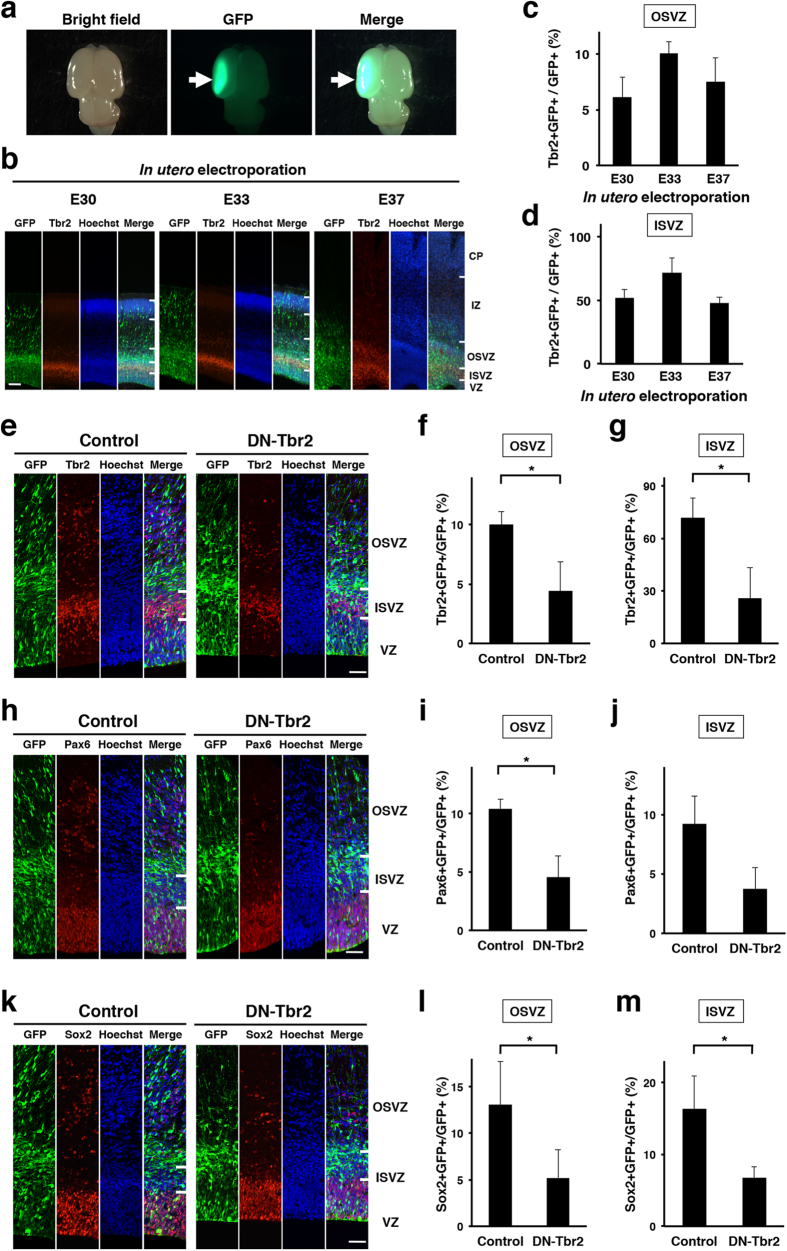
Tbr2 is required for the production of both IPCs and oRGs in the developing ferret cortex. (**a**) Dorsal views of the electroporated brain. Arrows indicate GFP-positive transfected areas. (**b**) IUE was performed at E30, E33 or E37, and coronal sections of the cerebral cortex were prepared 3 days later. The sections were stained with anti-Tbr2 antibody and Hoechst 33342. CP, cortical plate; IZ, intermediate zone; OSVZ, outer subventricular zone; ISVZ, inner subventricular zone; VZ ventricular zone. Scale bar = 100 μm. (**c**,**d**) Quantification of Tbr2-positive cells in GFP-positive transfected cells in (**b**). (**e**–**m**) pCAG-DN-Tbr2 and pCAG-GFP were introduced to the ferret cerebral cortex by using IUE at E33. Coronal sections of the cerebral cortex were prepared at E36 and stained with Hoechst 33342 plus either anti-Tbr2 antibody (**e**–**g**), anti-Pax6 antibody (**h**–**j**) or anti-Sox2 antibody (**k**–**m**). It should be noted that the anti-Tbr2 antibody used here recognizes endogenous Tbr2 but does not recognize transfected DN-Tbr2. (**e**) Tbr2 immunohistochemistry. The numbers of Tbr2-positive cells were reduced by DN-Tbr2 in the OSVZ and the ISVZ. (**f**,**g**) Quantification of GFP-positive cells co-localized with Tbr2 in the OSVZ (**f**) and in the ISVZ (**g**). The proportions of GFP-positive cells that co-localized with Tbr2 were significantly decreased by inhibiting Tbr2 function (n = 3 animals; *p < 0.05; Student’s *t*-test). (**h**) Pax6 immunohistochemistry. The numbers of Pax6-positive cells were reduced by DN-Tbr2 in the SVZ. (**i**,**j**) Quantification of GFP-positive cells co-localized with Pax6 in the OSVZ (**i**) and in the ISVZ (**j**). The proportions of GFP-positive cells that co-localized with Pax6 were significantly decreased by inhibiting Tbr2 function (n = 3 animals; *p < 0.05; Student’s *t*-test). (**k**) Sox2 immunohistochemistry. The numbers of Sox2-positive cells were reduced by DN-Tbr2 in the SVZ. (**l**,**m**) Quantification of GFP-positive cells co-localized with Sox2 in the OSVZ (**l**) and in the ISVZ (**m**). The proportions of GFP-positive cells that co-localized with Sox2 were significantly decreased by inhibiting Tbr2 function (n = 3 animals; *p < 0.05; Student’s *t*-test). Bars present mean ± SD. Scale bar = 50 μm.

To examine the roles of SVZ progenitors in cortical folding, we took an advantage of *in utero* electroporation (IUE), which has been widely used for expressing transgenes in the living rodent brain^29^,^30^,^31^,^32^,^33^. Recently, we successfully established an IUE technique for gyrencephalic carnivore ferrets, and our technique enabled us to manipulate gene expressions in the living ferret brain (Fig. 2a,b)^22^,^23^,^24^. To reduce SVZ progenitors in the ferret cerebral cortex, we utilized dominant-negative Tbr2 (DN-Tbr2), which is commonly used to inhibit Tbr2 activity in mice^34^,^35^. Because it was reported that Tbr2 was essential for directing RGs into IPCs in mice, and therefore IPCs were reduced in Tbr2 knockout mice^27^, we expected that DN-Tbr2 would reduce IPCs in the developing ferret cerebral cortex.

Before transfecting DN-Tbr2 into the ferret cerebral cortex using IUE, we examined at which age IUE should be performed to introduce transgenes into Tbr2-positive IPCs. This is because IUE in ferrets at different ages results in distinct cell populations being transfected^22^. We transfected pCAG-GFP, which expresses GFP under the control of the CAG promoter, using IUE at E30, E33 or E37, and examined how many GFP-positive cells expressed Tbr2 in the cerebral cortex 3 days later. Our quantitative analysis showed that more GFP-positive cells in the OSVZ and the ISVZ were Tbr2-positive when IUE was performed at E33 (Fig. 2c,d). We therefore carried out IUE at E33 to express DN-Tbr2 in IPCs in the following experiments.

To examine the effects of DN-Tbr2 on the production of IPCs, we expressed DN-Tbr2 in the developing ferret cortex using IUE at E33. Sections of the cerebral cortex were prepared at E36, and Tbr2 immunostaining was carried out (Fig. 2e). To selectively identify endogenous ferret Tbr2, we used anti-Tbr2 antibody, which recognizes endogenous ferret Tbr2 but does not detect transfected DN-Tbr2 (data not shown). As expected, the percentages of GFP-positive cells co-expressing Tbr2 were markedly reduced in the OSVZ (control, 10.0 ± 1.1%, 242 cells; DN-Tbr2, 4.5 ± 2.4%, 316 cells; p < 0.05; Student’s *t*-test) and in the ISVZ (control, 71.6 ± 11.6%, 180 cells; DN-Tbr2, 25.8 ± 17.6%, 216 cells; p < 0.05; Student’s *t-*test) (Fig. 2e–g). These results indicate that IPCs were significantly reduced by DN-Tbr2.

To examine the effect of DN-Tbr2 on the production of oRGs, the sections were stained with anti-Pax6 and anti-Sox2 antibodies (Fig. 2h,k). Interestingly, we found that the percentages of GFP-positive cells co-expressing Pax6 were also decreased in the OSVZ (control, 10.4 ± 0.8%, 191 cells; DN-Tbr2, 4.5 ± 1.8%, 374 cells; p < 0.05; Student’s *t*-test) and in the ISVZ (control, 9.2 ± 2.3%, 119 cells; DN-Tbr2, 3.8 ± 1.8%, 148 cells; p = 0.059; Student’s *t*-test) (Fig. 2i,j). Consistently, the percentages of GFP-positive cells co-expressing Sox2 were significantly decreased both in the OSVZ (control, 13.0 ± 4.7%, 229 cells; DN-Tbr2, 5.2 ± 3.0%, 306 cells; p < 0.05; Student’s *t*-test) and in the ISVZ (control, 16.4 ± 4.6%, 130 cells; DN-Tbr2, 6.7 ± 1.6%, 154 cells; p < 0.05; Student’s *t-*test) (Fig. 2l,m). These results demonstrate that two types of SVZ progenitors, IPCs and oRGs, were significantly reduced by inhibiting Tbr2 in the ferret cerebral cortex. These findings indicate that Tbr2 is essential not only for the production of IPCs, but also for that of oRGs.

### Induction of precocious neurogenesis by inhibition of Tbr2

**Figure 3:**
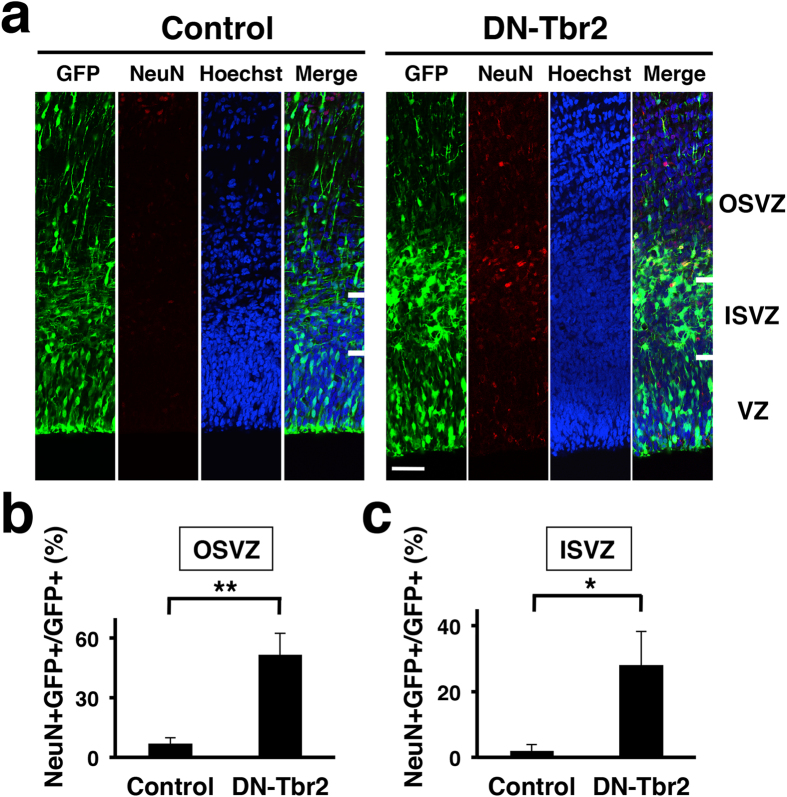
Inhibition of Tbr2 function induces precocious neurogenesis in the developing ferret cortex. pCAG-DN-Tbr2 and pCAG-GFP were introduced to the ferret cerebral cortex by using IUE at E33. Coronal sections of the cerebral cortex were prepared at E36 and stained with Hoechst 33342 and anti-NeuN antibody. (**a**) NeuN immunohistochemistry. Many NeuN-positive neurons were found in the SVZ of the DN-Tbr2-transfected cortex. Scale bar = 50 μm. (**b**,**c**) Quantification of GFP-positive cells co-localized with NeuN in the OSVZ (**b**) and in the ISVZ (**c**). The proportions of GFP-positive cells that co-localized with NeuN were markedly increased by inhibiting Tbr2 function (n = 3 animals; *p < 0.05; **p < 0.01; Student’s *t*-test). Bars present mean ± SD.

Because SVZ progenitors were reduced by DN-Tbr2, we hypothesized that DN-Tbr2-transfected RGs immediately differentiated into post-mitotic neurons without staying at the stages of IPCs and oRGs. To test this hypothesis, we stained the sections with anti-NeuN antibody (Fig. 3a), which recognizes post-mitotic neurons^26^,^27^,^36^. Interestingly, while NeuN was rarely found in the SVZ of the control cortex, we found abundant NeuN-positive cells in the SVZ of the DN-Tbr2-electroporated cortex (Fig. 3a). Consistently, our quantification demonstrated that the numbers of GFP-positive cells co-expressing NeuN were significantly increased in the OSVZ (control, 7.0 ± 2.9%, 218 cells; DN-Tbr2, 51.7 ± 10.7%, 269 cells; p < 0.01; Student’s *t*-test) and in the ISVZ (control, 2.0 ± 2.0%, 142 cells; DN-Tbr2, 28.0 ± 10.3%, 198 cells; p < 0.05; Student’s *t-*test) (Fig. 3b,c). These results indicate that DN-Tbr2 inhibits the production of SVZ progenitors and instead induces precocious neurogenesis in the SVZ.

### SVZ progenitors are indispensable for cortical folding

**Figure 4:**
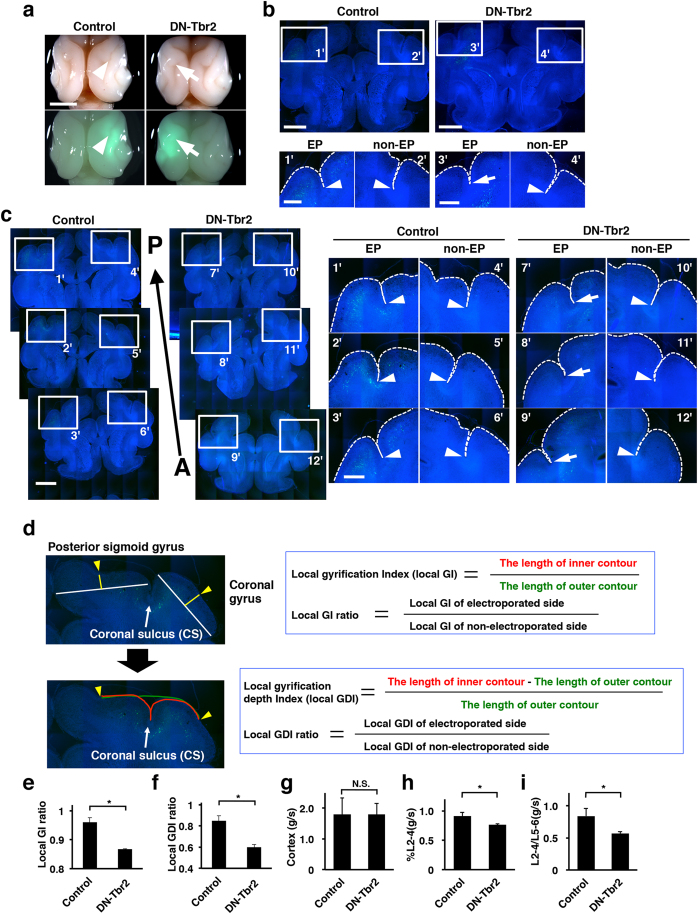
SVZ progenitors were indispensable for cortical folding. IUE was performed at E32-E33, and the brain was prepared at P16. (**a**) Dorsal macroscopic images. Upper panels show bright field images, and lower panels show bright field images merged with GFP images. Although the coronal sulcus was clearly formed on the control brain (arrowheads), it was obscure on the DN-Tbr2-electroporated brain (arrows). Scale bar = 5 mm. (**b**) Coronal sections of the brain stained with Hoechst 33342. Broken lines indicate the surface of the brain. Note that the depth of the coronal sulcus was markedly shallower on the DN-Tbr2-electroporated side of the cortex (3′, arrow) compared with that on the GFP-electroporated cortex (1′, arrowhead) and the non-electroporated side of the cortex (2′ and 4′, arrowheads). (**c**) Serial coronal sections of the brain stained with Hoechst 33342. A, anterior. P, posterior. Broken lines indicate the surface of the brain. (**d**) Quantification procedures of gyrification. (**e**) Quantification of the local GI ratio. The local GI ratio was significantly reduced by DN-Tbr2 (n = 3 animals; *p < 0.05; Welch’s *t*-test). Bars present mean ± SEM. (**f**) Quantification of the local GDI ratio. The local GDI ratio was significantly reduced by DN-Tbr2 (n = 3 animals; *p < 0.05; Student’s *t*-test). Bars present mean ± SEM. (**g**) The cortical thickness of the posterior sigmoid gyrus divided by that of the coronal sulcus. The thickness(g/s) values were not affected by DN-Tbr2. (n = 3 animals; p > 0.05; Student’s *t*-test; N.S. not significant). Bars present mean ± SD. (**h**) The thickness of layer 2–4 relative to the cortical thickness (%L2-4) was measured, and the ratio of %L2-4 in the posterior sigmoid gyrus to that in the coronal sulcus [%L2-4(g/s)] was calculated (n = 3 animals; *p < 0.05; Student’s *t*-test). Bars present mean ± SD. (**i**) The thickness of layer 2–4 relative to that of layer 5–6 (L2-4/L5-6) was measured, and the ratio of L2-4/L5-6 in the posterior sigmoid gyrus to that in the coronal sulcus [L2-4/L5-6 (g/s)] was calculated (n = 3 animals; *p < 0.05; Student’s *t*-test). Bars present mean ± SD.

To examine the role of SVZ progenitors in cortical folding, we expressed DN-Tbr2 in the ferret cerebral cortex using IUE at E33, and the brains were prepared at postnatal day 16 (P16), when cortical folds are formed (Fig. 4a). We found that the sulcus on the GFP-positive transfected cortical area was much shallower in the DN-Tbr2-transfected cortex (Fig. 4a, arrows) compared to that in the control cortex (Fig. 4a, arrowheads).

To examine the effect of DN-Tbr2 on the depth of sulci, coronal sections of the electroporated brains were prepared and stained with Hoechst 33342 (Fig. 4b). Interestingly, we found that the depth of the sulcus in the cortical area electroporated with DN-Tbr2 (Fig. 4b,3′, arrow) was markedly shallower than that in the GFP-electroporated cortex (Fig. 4b,1′, arrowhead) and those in the contralateral non-transfected sides of each cortex (Fig. 4b,2′,4′, arrowheads). Our experiments using serial sections of the ferret brain consistently showed similar results (Fig. 4c). To quantify these results, we defined the local gyrification index (local GI), which is the length of the inner complete contour (i.e. the pial surface) (Fig. 4d, red line) divided by that of the outer contour of the cortex (Fig. 4d, green line) ranging from the top of the posterior sigmoid gyrus to that of the coronal gyrus (Fig. 4d, yellow arrowheads). To minimize the variation of the local GI values depending on the position of coronal sections in the brain, the local GI values on the transfected side and the contralateral control side of the cerebral cortex in the same brain sections were measured, and the former was divided by the latter (local GI ratio) (Fig. 4d). The local GI ratio would be 1 if the depth of cortical folding was the same between the transfected side and the other side, and would be smaller than 1 if cortical folding was suppressed by genetic manipulation. Consistent with our macroscopic observations (Fig. 4a–c), we found that the local GI ratio was significantly smaller in the DN-Tbr2-transfected cortex than in the GFP-transfected control cortex (control, 0.96 ± 0.016; DN-Tbr2, 0.87 ± 0.0024; p < 0.05, Welch’s *t*-test) (Fig. 4e), suggesting that cortical folding is impaired in the DN-Tbr2-transfected cortex.

We also defined another index, the local gyrification depth index (local GDI), which represents the depth of the coronal sulcus (Fig. 4d). The local GDI values were measured around the coronal sulcus on the transfected side and the contralateral control side of the cerebral cortex. As in the case of the local GI ratio, to minimize the variation of the local GDI values depending on the position of coronal sections in the brain, the local GDI values on the transfected side and the contralateral control side of the cerebral cortex in the same brain section were measured, and the former was divided by the latter (local GDI ratio) (Fig. 4d). The local GDI ratio would be 1 if the depths of the coronal sulcus were the same between the transfected side and the other side, and would be smaller than 1 if the depth was shallower on the transfected side. Consistent with the results obtained with the local GI values, we found that the local GDI ratios were markedly smaller in the DN-Tbr2-transfected cortex than in the GFP-transfected control cortex (control, 0.85 ± 0.048; DN-Tbr2, 0.60 ± 0.024; p < 0.05, Student’s *t*-test) (Fig. 4f). These results indicate that Tbr2 is required for cortical folding during development. Taken together with our results showing that SVZ progenitors are decreased by DN-Tbr2, our findings suggest that SVZ progenitors are indispensable for gyrification during development.

Given the regional difference in the abundance of Tbr2-positive cells (Fig. 1), we hypothesized that DN-Tbr2 more severely affected gyri rather than sulci. We first compared the thickness of the cerebral cortex between gyri and sulci at P16. To examine the regional differences in the effect of DN-Tbr2, we calculated the ratio of the cortical thickness of the gyrus to that of the sulcus [thickness(g/s)]. The thickness(g/s) values were almost the same between the control GFP-electroporated cortex and the DN-Tbr2 electroporated cortex (control, 1.79 ± 0.53; DN-Tbr2, 1.79 ± 0.35; p > 0.05, Student’s *t*-test) (Fig. 4g).

It has been proposed that the increase in upper layer neurons of the developing cortex is responsible for the formation of the gyrus^4^,^7^,^9^. We therefore measured the thickness of layer 2–4 relative to the cortical thickness (%L2-4), and calculated the ratio of %L2-4 in the gyrus to that in the sulcus [%L2-4(g/s)]. Layer 2–4 and layer 5–6 of the ferret cerebral cortex were identified using Hoechst 33342 staining, and were confirmed by NeuN, Ctip2 and FoxP2 immunohistochemistry (Supplementary Fig. 2). Interestingly, we found that %L2-4(g/s) significantly decreased in the DN-Tbr2-transfected cortex (control, 0.91 ± 0.064; DN-Tbr2, 0.76 ± 0.019; p < 0.05, Student’s *t*-test) (Fig. 4h), suggesting that DN-Tbr2 preferentially reduced upper layers in the gyrus rather than those in the sulcus. This observation is consistent with recent reports that SVZ progenitors were more abundantly distributed in the prospective gyrus^4^,^25^,^28^.

In addition to %L2-4(g/s), we newly defined L2-4/L5-6(g/s). The thickness of layer 2–4 and that of layer 5–6 were measured, and the ratio of these values was calculated (L2-4/L5-6). To examine the regional difference of the effect of DN-Tbr2, the L2-4/L5-6 value in the gyrus was divided by that in the sulcus [L2-4/L5-6(g/s)]. Consistent with the %L2-4(g/s) values, the L2-4/L5-6(g/s) values were significantly decreased in DN-Tbr2-transfected cortices (control, 0.84 ± 0.12; DN-Tbr2, 0.57 ± 0.027; p < 0.05, Student’s *t*-test) (Fig. 4i). Taken together with our results of %L2-4(g/s), our findings indicate that DN-Tbr2 preferentially reduced upper layers in the gyrus rather than those in the sulcus. Our findings are consistent with the idea that regional differences in the abundance of SVZ progenitors are crucial for gyrification. Our findings that regional differences in the abundance of SVZ progenitors were more obvious later during development when upper layer neurons are formed (Fig. 1) also support this idea.

## Discussion

Here we have shown regional differences in the abundance of SVZ progenitors in the ferret cerebral cortex even before gyrus formation starts. Using our IUE procedure optimized to express genes of interest into SVZ progenitors, we demonstrated that the inhibition of Tbr2 significantly reduced not only IPCs but also oRGs. Furthermore, we uncovered that the reduction of SVZ progenitors resulted in the attenuation of cortical folding and reduced upper layers especially in the gyrus. Taken together, our findings indicate that SVZ progenitors are crucial for cortical folding in gyrencephalic mammals.

Although it has been proposed that the acquisition of cortical folding during evolution resulted from increased proliferation of neural progenitor cells in the cerebral cortex^4^,^5^,^6^,^7^,^8^,^16^, the roles of SVZ progenitors in cortical folding of gyrencephalic mammals during development were not well understood. Furthermore, recent reports proposed that the increase of SVZ progenitors is dispensable for cortical folding^20^,^21^. In contrast, our findings using ferrets provided firm *in vivo* data indicating that the increase of SVZ progenitors is crucial for cortical folding during development. Consistently, we recently demonstrated that ectopic expression of FGF8 in the ferret cerebral cortex led to an increase in SVZ progenitors and polymicrogyria, suggesting that increased SVZ progenitors may underlie the formation of additional gyri^24^. Since the abundance of SVZ progenitors shows regional differences in the ferret cerebral cortex, these regional differences are likely to be a key for distinguishing where gyri and sulci are formed. It is plausible that regions with abundant SVZ progenitors would be gyri, whereas those with fewer SVZ progenitors would be sulci. Our observations strongly support the idea that regional differences in the proliferation of SVZ progenitors play a crucial role in cortical gyrification^4^,^11^,^25^,^28^. Because suppression of Tbr2 function decreased both IPCs and oRGs, it was unclear which play more important roles in cortical gyrification. Therefore, it would be intriguing to distinguish the roles of IPCs and oRGs in cortical gyrification. We also showed that upper layers in the cerebral cortex were more affected in gyri than in sulci by DN-Tbr2. It seems likely that regions with abundant SVZ progenitors would produce much more upper layer neurons, resulting in the formation of gyri. Previously, it was proposed that an increase in upper layer neurons is responsible for the formation of gyri^4^,^7^,^9^. Our findings are consistent with this hypothesis. It would be intriguing to further investigate the molecular mechanisms underlying the regional differences in the abundance of SVZ progenitors in the cerebral cortex of gyrencephalic mammals. Recently, it was reported that progenitors in the ferret cortex exhibited longer cell cycles than those in the mouse cortex and that the duration of S-phase is different among the various progenitor types in the developing ferret cortex^37^. It would be important to investigate regional differences in cell cycle parameters in the developing ferret cortex because it is possible that they lead to regional differences in the abundance of SVZ progenitors.

We showed that inhibition of Tbr2 significantly reduced IPCs and induced precocious neurogenesis in the SVZ (Figs 2 and 3). This is consistent with a previous report showing that Tbr2 is essential for IPC production in the mouse cortex^27^. Interestingly, the inhibition of Tbr2 also resulted in the reduction of Pax6- and Sox2-positive cells in the SVZ (Fig. 2). It seems possible that Tbr2 is required for the production of both IPCs and oRGs cell-autonomously. Tbr2 could be transiently expressed when RGs are differentiating into oRGs, and could be indispensable for their differentiation into oRGs. Alternatively, it is also possible that Tbr2 directly regulates the production of IPCs, and IPCs have some non-cell-autonomous effects on oRG production. It would be important to investigate the detailed mechanisms underlying the development of IPCs and oRGs in the cerebral cortex of gyrencephalic mammals.

We demonstrated that DN-Tbr2 successfully reduced SVZ progenitors in the developing ferret cerebral cortex. It seemed likely that DN-Tbr2 reduced SVZ progenitors by suppressing endogenous ferret Tbr2 because the amino acid sequence of mouse Tbr2 is highly homologus to that of ferret Tbr2 (86%) (Supplementary Fig. 3). Importantly, the amino acid sequence of the T-box DNA-binding domain of mouse Tbr2 is 100% identical to that of ferret Tbr2 (Supplementary Fig. 3). On the other hand, we cannot exclude the possibility that T-box transcriptional factors other than Tbr2 are also involved in the phenotypes induced by DN-Tbr2. Further investigations would be necessary for uncovering the entire picture of the mechanisms mediated by Tbr2 in gyrencephalic mammals.

Although our results clearly demonstrated the importance of SVZ progenitors in cortical folding, cortical folding was not completely abolished by the reduction of SVZ progenitors. One possible reason is that a complete inhibition of the production of all SVZ progenitors is necessary to eliminate cortical folding. Even though we optimized the efficiency of electroporation, SVZ progenitors were not completely eliminated (Fig. 2). We performed IUE at E33 in this study, but progenitors are also produced both earlier and later than E33 (Fig. 2), and these progenitors were not transfected with IUE at E33. Transfecting all SVZ progenitors with DN-Tbr2 would help uncovering this issue, although it is technically difficult at this moment. Another possibility is that, in addition to SVZ progenitors, other mechanisms also mediate cortical folding. This seems likely because the amounts of SVZ progenitors are not simply correlated with cortical folding^20^,^21^. Other mechanisms such as mechanical tension produced by axonal projections, radial glial scaffolding and ventricular surface expansion have been proposed to underlie cortical folding^4^,^5^,^6^,^7^,^8^,^9^,^10^,^11^,^12^. It would be important to uncover the entire picture of the mechanisms underlying cortical gyrification during development. The recently established molecular biological methods for ferrets would be powerful tools to investigate this question^22^,^23^,^25^,^38^,^39^,^40^,^41^.

## Methods

### Animals

Normally pigmented, sable ferrets (*Mustela putorius furo*) were purchased from Marshall Farms (North Rose, NY). Ferrets were maintained as described previously^38^,^39^,^42^. The day of conception and that of birth were counted as embryonic day 0 (E0) and postnatal day 0 (P0), respectively. All procedures were approved by the Animal Care Committee of Kanazawa University and the University of Tokyo and were performed in accordance with protocols approved by them.

### *In utero* electroporation (IUE) procedure for ferrets

By modifying the procedure for IUE for mice, we recently established a procedure of IUE to express transgenes in the ferret brain^22^,^23^. Briefly, pregnant ferrets were anesthetized with sodium pentobarbital, and their body temperature was monitored and maintained using a heating pad. The uterine horns were exposed and kept wet by adding drops of PBS intermittently. The location of embryos was visualized with transmitted light delivered through an optical fiber cable. The pigmented iris was visible, and this enabled us to assume the location of the lateral ventricle. Approximately 2–5 μl of DNA solution was injected into the lateral ventricle at the indicated ages using a pulled glass micropipette. Each embryo within the uterus was placed between tweezer-type electrodes with a diameter of 5 mm (CUY650-P5; NEPA Gene, Japan). Square electric pulses (50–100 V, 50 ms) were passed 5 times at 1-second intervals using an electroporator (ECM830, BTX). The wall and skin of the abdominal cavity were sutured, and the embryos were allowed to develop normally.

### Plasmids

pCAG-GFP was described previously^32^. DN-Tbr2 was kindly provided by Dr. Steven L. Reiner (Columbia University). Briefly, DN-Tbr2 was made by fusing C-terminal truncated mouse Tbr2 (amino acid 1-522) to the repression domain of *Drosophila* engrailed (amino acid 1-299)^34^,^35^ and was subcloned into the *BamHI*-*XhoI* site of a pCAG plasmid vector, yielding pCAG-DN-Tbr2. Plasmids were purified using the Endofree Plasmid Maxi Kit (Qiagen, Valencia, CA). Prior to IUE procedures, plasmid DNA was diluted to 2.5 mg/ml in PBS, and Fast Green solution was added to a final concentration of 0.5% to monitor the injection. For co-transfection, a mixture of pCAG-GFP and pCAG-DN-Tbr2 was used.

### Homology comparison

Comparison of the amino acid sequence of mouse Tbr2 (NP_034266.2) and that of putative ferret Tbr2 (XP_004754427.1) was conducted using the ClustalW function of MacVector software.

### Preparation of sections

Preparation of sections was performed as described previously with slight modifications^39^,^43^. Briefly, ferrets were deeply anesthetized with pentobarbital and transcardially perfused with 4% paraformaldehyde (PFA), and the brains were dissected. Then, the brains were cryoprotected by three-day immersion in 30% sucrose and embedded in OCT compound. Sections of 50 μm thickness were prepared using a cryostat.

### Immunohistochemistry

Immunohistochemistry was performed as described previously with slight modifications^39^,^43^,^44^. Coronal sections (50 μm) were made using a cryostat, permeabilized with 0.1–0.5% Triton X-100/PBS, and incubated overnight with primary antibodies, which included anti-Tbr2 antibody (Abcam, ab23345), anti-Sox2 antibody (R&D Systems, AF2018), anti-Pax6 antibody (Covance, PRB-278P), anti-NeuN antibody (Chemicon, MAB377), anti-FOXP2 antibody (Atlas, HPA000382), anti-Ctip2 antibody (Abcam, ab18465) and anti-GFP antibodies (Nacalai tesque, Japan, 04404-26; Medical & Biological Laboratories, Japan, 598). After incubation with secondary antibodies and Hoechst 33342, the sections were washed and mounted.

### Cell counting

Confocal images were acquired using an Olympus FV10i microscope (optical section = 1.4 μm). Images were collected from sections stained with anti-Tbr2, Pax6, Sox2 and NeuN antibodies and Hoechst 33342. The number of GFP-positive cells co-localized with Tbr2, Pax6, Sox2 and NeuN were manually counted using the “cell counter” function of ImageJ software. We only counted the number of GFP-positive cells with nuclei which were visualized with Hoechst 33342 staining. The numbers of positive cells were counted using three different animals. As described previously^16^, the cell-dense layer next to the VZ was identified as the ISVZ and the cell-sparse layer between the ISVZ and the IZ was identified as the OSVZ. In addition, the border between the ISVZ and the OSVZ was also identified by the inner fiber layer (IFL), visualized as GFP-positive fibers in the OSVZ^23^.

### Calculation of the local GI ratio and the local GDI ratio

The calculation of the local GI ratio and the local GDI ratio in electroporated ferrets was performed on 50 μm thick serial coronal sections. Coronal sections containing both the anterior part of the striatum and electroporated cells spanning from the posterior sigmoid gyrus to the coronal gyrus were used for quantification (Fig. 4c). The sections were stained with Hoechst 33342, and tiling images of the whole cerebral cortex were acquired using a BZ-9000 microscope (Keyence).

We first determined the area for the quantification. In both the posterior sigmoid gyrus and the coronal gyrus, we drew lines at the widest points (Fig. 4c, white lines). Then, we drew lines perpendicular to the white lines at the midpoint of white lines (Fig. 4c, yellow lines). Points where the yellow lines intersect with brain surface were marked (Fig. 4c, yellow arrowheads), and then the local GI values and the local GDI values were calculated using the area between the yellow arrowheads, as shown in Fig. 4c (green line and red line). To minimize the variation of the local GI and the local GDI values depending on the position of coronal sections in the brain, the local GI and the local GDI values of the non-electroporated hemisphere in the same section were also calculated, and the local GI ratio and the local GDI ratio were defined as follows (Fig. 4c).


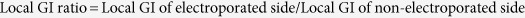



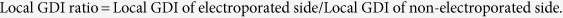


### Quantification of cortical thickness and cell densities

Coronal sections, containing both the anterior part of the striatum and electroporated cells spanning from the posterior sigmoid gyrus to the coronal gyrus were used for quantification. The sections were stained with Hoechst 33342, and confocal images were acquired using an Olympus FV10i microscope. The cortical thickness was measured at the coronal sulcus and the posterior sigmoid gyrus using ImageJ software. Cortical layers were identified by Hoechst 33342 staining and immunohistochemistry using anti-NeuN, Ctip2 and FOXP2 antibodies.

### Quantification of the distribution of SVZ progenitors

Coronal sections containing the dorsal telencephalon were stained with anti-Tbr2 antibody and anti-Pax6 antibody. Images were acquired using an Axioimager A1 microscope (Carl Zeiss). After background signals were subtracted, optical densities within rectangles (Fig. 1b,b1–b4,f,f1–f3, white boxes), which extended from the ventricular surface to the pial surface, were measured using ImageJ software. In the plot of Tbr2 signals (Fig. 1c), the first peak from the ventricular surface corresponded to the ISVZ, and the 2nd peak corresponded to the OSVZ. Signal intensities in the peaks of the OSVZ were measured (Fig. 1d). In the plot of Pax6 signals (Fig. 1g), the first peak from the ventricular surface corresponded to the VZ, and the 2nd peak corresponded to the SVZ. Signal intensities in the peaks of the SVZ were measured (Fig. 1h).

### Statistical analyses

Statistical analyses were performed using Statcel2 software (OMS Publishing, Japan) and R software. To assess statistical significance, the Kolmogorov-Smirnov test was performed, and p values were determined by the unpaired Student’s *t*-test or the Welch’s *t*-test. “n” means the number of animals.

## Additional Information

**How to cite this article**: Toda, T. *et al.* An essential role of SVZ progenitors in cortical folding in gyrencephalic mammals. *Sci. Rep.* **6**, 29578; doi: 10.1038/srep29578 (2016).

## Supplementary Material

Supplementary Information
