# Supplementary material for: An essential role of SVZ progenitors in cortical folding in gyrencephalic mammals
Source: Sci Rep. 2016 Jul 12;6:29578. doi: 10.1038/srep29578 (PMC4941724; doi:10.1038/srep29578)
Supplement: Supplementary Information [file srep29578-s1.pdf]

Supplementary Information

An essential role of SVZ progenitors in cortical folding in gyrencephalic mammals

Tomohisa Toda, Yohei Shinmyo, Tung Anh Dinh Duong, Kosuke Masuda and Hiroshi Kawasaki

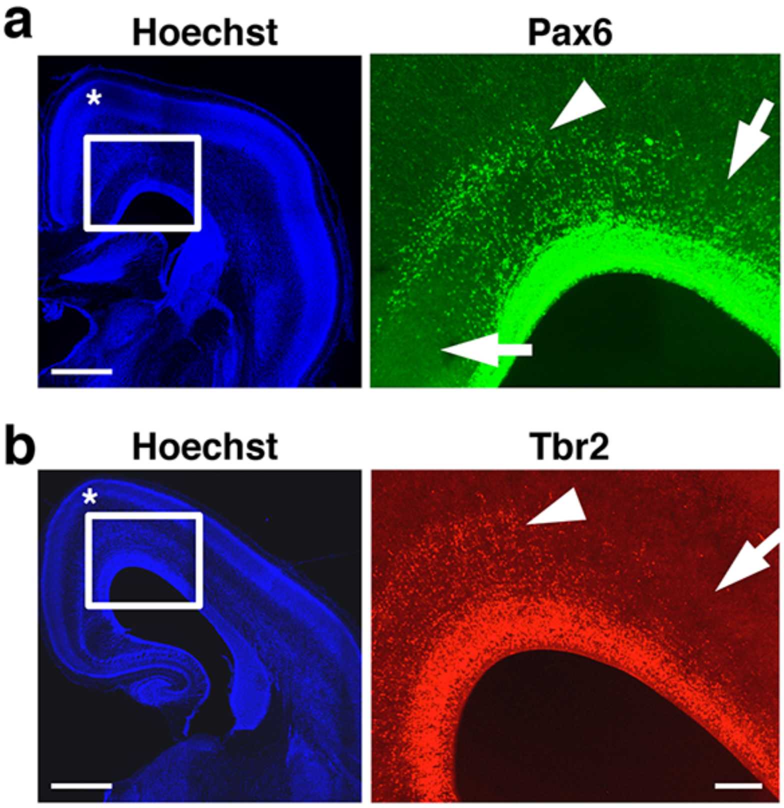

Supplementary Figure 1. Expression patterns of Tbr2 and Pax6 in the ferret cerebral cortex at P6. (**a**) Coronal sections of the cerebral cortex were prepared at P6 and stained with Hoechst 33342 plus either anti-Pax6 antibody (**a**) or anti-Tbr2 antibody (**b**). Pax6 and Tbr2 images within the boxes are shown on the right. Asterisks indicate prospective areas of the lateral gyrus. Note that Tbr2 and Pax6 are abundantly expressed in the prospective areas of the lateral gyrus (arrowheads) rather than other areas (arrows). Scale bars = 1 mm (left), 200 μm (right).

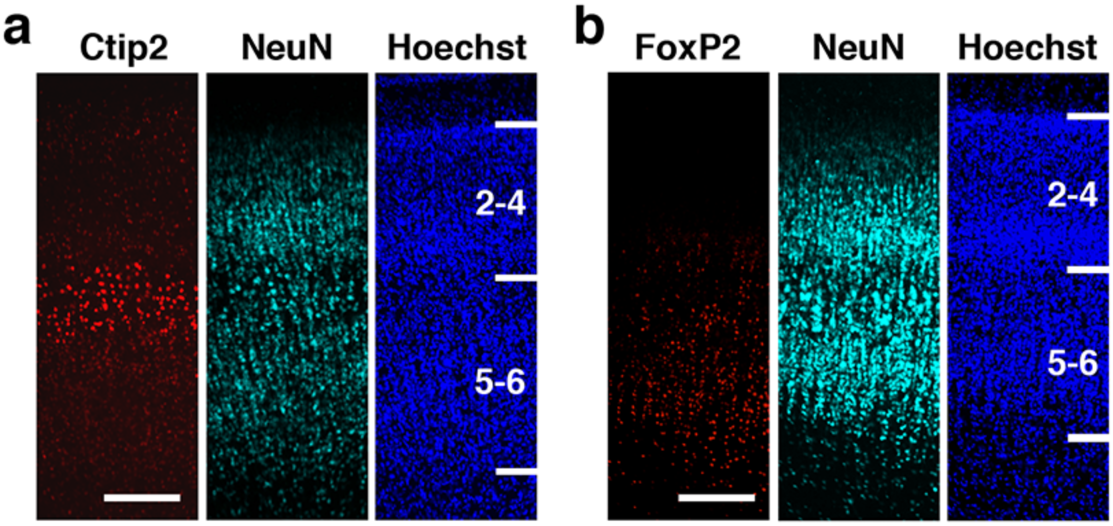

Supplementary Figure 2. Expression of layer-specific markers in the developing ferret cortex. Coronal sections of the ferret cerebral cortex were prepared at P16 and stained with Hoechst 33342 and anti-NeuN antibody plus either anti-Ctip2 antibody (**a**) or anti-FOXP2 antibody (**b**). The numbers indicate layers of the cerebral cortex. Scale bars = 200 μm.

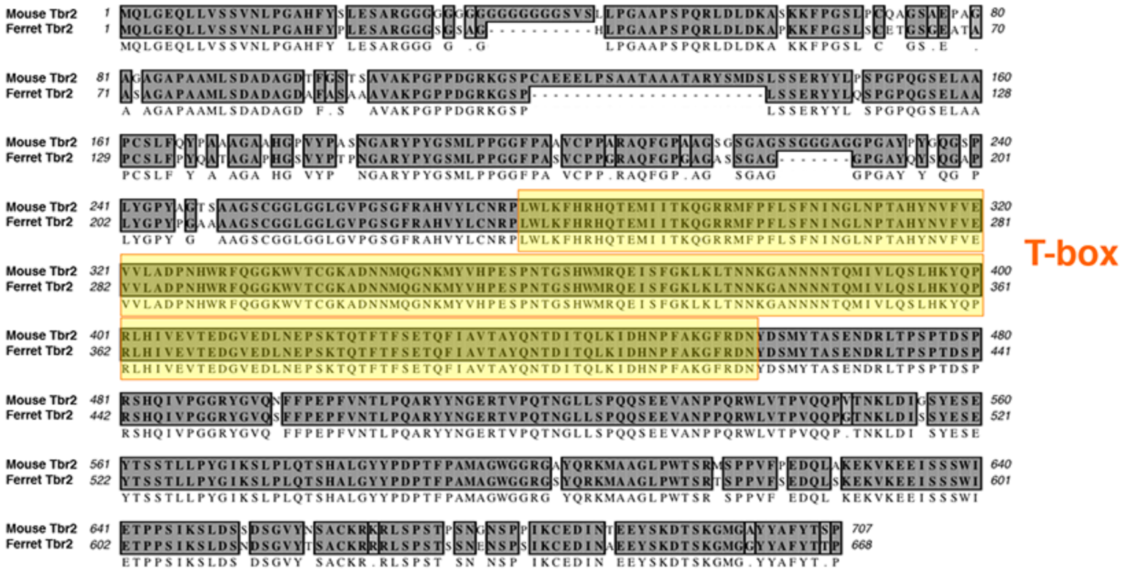

Supplementary Figure 3. Comparison of the amino acid sequence of mouse Tbr2 and that of ferret Tbr2. The yellow area indicates the T-box domain. Note that the amino acid sequence of the T-box domain of mouse Tbr2 is 100% identical to that of ferret Tbr2.
